# Academy of Sciences, Paris

**Published:** 1889-11

**Authors:** A. Dagenais

**Affiliations:** Buffalo, N. Y.


### Translations.

### ACADEMY OF SCIENCES, PARIS.

From Progrès Médical. By A. DAGENAIS, M. D., Buffalo, N. Y.

M. VERNEUIL called, once more, attention to the pathogenic properties of microbes contained in malignant tumors. The tissue of malignant neoplasms (cancers, sarcoma, epithelioma, etc.,) may be invaded, at any time, by different microbes, of which we cannot, for certain, yet determine the origin, gender, or number. This invasion, of which the causes and mechanism are equally unknown, may remain more or less concealed; but, also, in certain cases, it may bring in the evolution and nutrition of these tumors many modifications, as rapid growth, softening, and ulceration. These microbes are not always met with in all the different neoplasms, neither in all the neoplasms of the same kind—not even in all the part of the neoplasm already invaded. They are not found, for instance, in all the lipoma, nor pure fibroma, neither in sarcoma or incipient cancers, with slow progress, and covered with healthy skin; on the contrary, they are found in the soft and ulcerated neoplasms. Beside the irritating effect which they manifest locally on the tissue of the tumor invaded, these microbes possess other pathogenic properties which interest the old economy. When they are contained in a tumor of rapid growth, or of softening tendency, they will give rise to a fever more or less intense and irregular. Moreover, during the ablation of a tumor which contains these microbes, they may, if mixed with the softened parts surrounding the tumor, contaminate, infect, and inoculate, which will cause a septicemic fever capable of producing death. The knowledge of this last fact goes to plead in favor of the ablation of malignant tumors, and at the same time dictates to the surgeon certain preventative measures during and after the extirpation of tumors infected with microbes.

